# Intestinal Organoids as a Tool for Inflammatory Bowel Disease Research

**DOI:** 10.3389/fmed.2019.00334

**Published:** 2020-01-17

**Authors:** Hamish C. K. Angus, A. Grant Butt, Michael Schultz, Roslyn A. Kemp

**Affiliations:** ^1^Department of Microbiology and Immunology, University of Otago, Dunedin, New Zealand; ^2^Department of Physiology, University of Otago, Dunedin, New Zealand; ^3^Department of Medicine, University of Otago, Dunedin, New Zealand

**Keywords:** Inflammatory Bowel Disease, immunology, organoid, T cells, cytokines, regulation, diagnostics

## Abstract

Inflammatory Bowel Diseases (IBD) are difficult to model as freshly acquired tissues are short-lived, provide data as a snapshot in time, and are not always accessible. Many patients with IBD are non-responders to first-line treatments, and responders are prone to developing resistance to treatment over time—resulting in reduced patient quality of life, increased time to remission, and potential relapse. IBD is heterogenous and we are yet to fully understand the mechanisms of disease; thus, our ability to diagnose and prescribe optimal treatment remains ineffective. Intestinal organoids are derived from patient tissues expanded *in vitro*. Organoids offer unique insight into individual patient disease and are a potential route to personalized treatments. However, organoid models do not contain functional microbial and immune cell components. In this review, we discuss immune cell subsets in the context of IBD, and the requirement of immune cell and microbial components in organoid models for IBD research.

## Introduction

Intestinal organoids are a three-dimensional *in vitro* model of the human intestinal epithelium that allow for robust, patient specific *in vitro* research of the development and properties of the intestinal epithelium. The prevalence of Inflammatory Bowel Diseases (IBD) is rapidly increasing across both developed and developing countries (1). IBD, such as Crohn's disease (CD) and Ulcerative colitis (UC), affects up to 0.5% of people in the Western world (1). Due to a lack of patient specificity and knowledge of disease mechanisms, successful treatment of these diseases remains difficult. Frontline IBD treatments have limited efficacy in large groups of patients. For example, Infliximab, a biologic anti-tumor necrosis factor (TNF) antibody treatment, is only effective in 60–87% of patients, 23–46% of whom become secondary non-responders within 5 years (2). Mechanisms of IBD are yet to be elucidated and are difficult to pinpoint in individual patients.

In this review, we explore the potential benefits and limitations of intestinal organoid cultures for immunological research in IBD. The terminology for organoids is complex and is used interchangeably. In this review, we refer to an intestinal “organoid” as a self-organizing, self-renewing, multicell-complex nominally derived from intestinal crypt Leucine-rich repeat-containing G-protein coupled receptor 5 (Lgr5)+ stem cells excised from primary tissue, human or murine (3). This definition is distinct from organoids derived from induced pluripotent stem cells (iPSCs), which contain both an epithelial and mesenchymal component (4). Primary intestinal crypt stem cells, when grown in suitable matrix and media, organize themselves into three-dimensional epithelial structures, exhibiting genetic and physiological similarities to their organ of origin.

Intestinal organoid models are the result of stem cell research and still lack standardized methods to include the intestinal microbiota and immune cells of the lamina propria. IBD is the result of a complex interplay between the intestinal epithelial barrier (IEB), the immune system, and the microbiota (5). The addition of a viable immune system to the organoid model, as well as a microbiota, may allow for mechanistic studies of IBD. Here, we discuss intestinal organoid models and their relevance and requirement for the development of a biologically accurate *in vitro* model of intestinal inflammatory diseases, focusing on intestinal immune cells.

## The Immune System in IBD

IBD is potentially only an umbrella term for different diseases, most of which have not yet been accurately described. Identified mechanisms that can lead to IBD include: loss of immune tolerance to commensal bacteria, inflammatory and suppressive immune cell defects, polymorphisms in pattern-recognition receptor genes (e.g., *NOD2*), defects in autophagy, and tight junction dysregulation and defects (6, 7). Each mechanism is different; however, all have one trait in common—each result in varying degrees of disruption of the IEB, their associated tight junction proteins, and the overlaying mucus layer. On the basolateral side of the IEB is the lamina propria, a tightly regulated region of the intestine, containing a large immune population on the basolateral border of the IEB. This immune population is located in the lamina propria to detect and clear viral, fungal, and bacterial migrants from the lumen.

Cells of the IEB contain Toll-like receptors (TLRs) that can induce production of inflammatory or anti-inflammatory responses to foreign materials (8). For example, binding of TLR9—a membrane-bound protein complex, stimulated an anti-inflammatory tolerogenic response upon binding of bacterial unmethylated CpG dinucleotides on the apical surface (lumen) of enterocytes, whereas binding of CpG by basolateral (lamina propria) TLR9 stimulated a pro-inflammatory response (8). In a homeostatic scenario, the polarized nature of the TLR response of the epithelial cells allows protection against invaders that breach the barrier, without an excessive response to the luminal microbiota; however, the disruption of the IEB in IBD patients allows greater migration of luminal contents into the lamina propria (9). This is one example of a pathway that can result in the establishment of a positive feedback loop of epithelial inflammation. Immune cells detect the unwanted foreign presence in the lamina propria and mount an immune response. Inflammatory cytokines, such as IL-6, IL-17A, IL-17F, interferon (IFN)γ, and TNF are produced by resident immune cells (10). While this immune response can contribute to increased epithelial turnover and pathogen clearance, it can also generate off-target cellular damage, further increasing IEB permeability.

An in-depth analysis on every immune cell type and their associations with disease is beyond the scope of this review. However, we will discuss some immune cell subsets frequently associated with disease status in IBD patients.

### Antigen Presenting Cells (APCs)

Antigen presenting cells, such as dendritic cells (DCs) and macrophages, are important sentinels of gut microbial and dietary antigens. DCs are situated in the lamina propria in gut-associated lymphoid tissues, such as Peyer's patches and intestinal draining lymph nodes. DCs have long dendrite projections that can sample luminal antigens via paracellular spaces. DCs provide T cells with pro- or anti-inflammatory signals. There are two major types of DC found in the gut, CD103+ and CD103– DCs. CD103 is a marker of immune cell residency; its ligand, E-cadherin, is a surface protein found on intestinal epithelial cells. CD103+ DCs, broadly speaking, have a suppressive immune capacity, presenting antigen to lamina propria T cells and driving regulatory T cell (Treg) differentiation. CD103– DCs in the gut can prime Th1 and Th17 T cells but are less commonly associated with gut tissues due to the absence of the CD103 integrin (11). DCs in the gut respond to the presence of retinoic acid and transforming growth factor-β (TGF-β), secreted by intestinal epithelial cell interactions with luminal microbes (12). This DC-epithelial crosstalk drives intestinal tolerance by inducing a suppressive DC phenotype. Patients with IBD have been reported to have lower frequencies of CD103+ DCs in both inflamed and non-inflamed tissues and have reduced ability to induce Treg differentiation, compared to healthy controls (13).

### Regulatory T Cells (Tregs)

Tregs are critical in the regulation of the intestinal environment. Peripheral Tregs express T cell receptors (TCRs) specific for self-antigens, in contrast to intestinal Tregs, which can express TCRs specific for microbial antigens. Tregs are abundant in the intestinal mucosa and are also modulated directly by bacterial antigens. For example, *Faecalibacterium prausnitzii* and *Bacteroides fragilis*, both common gut commensals, induced Treg activation and differentiation via the surface proteins, microbial anti-inflammatory molecule (MAM) and polysaccharide A (PSA), respectively (14, 15). Both *F. prausnitzii* and *B. fragilis* are often missing or present at low abundancy in patients with IBD (16). Both MAM and PSA prevented dextran sulfate sodium (DSS)- and 2,4,6-trinitrobenzenesulfonic acid (TNBS)-induced colitis in murine models, reducing Th1, Th17, and Th2 immune responses, and promoting Treg production of IL-10 and TGF-β (14, 15). TGF-β and IL-10 are suppressive cytokines, primarily produced by Tregs and tolerogenic DCs.

In the context of intestinal epithelial cells, TGF-β is an inducer of epithelial-mesenchymal transition (EMT). EMT is a process in which cells of the epithelial barrier lose their cellular polarity and transition into a mesenchymal phenotype (17). This process is a natural part of wound healing. In an immune context, TGF-β is recognized by TGFβ-receptors I and II, which initiate downstream signaling through SMAD1/3/4/6/7, resulting in suppression of inflammatory responses and induction of CD4+ T cell differentiation into Tregs. Murine models deficient in TGF-β or specifically blocked in T cell-TGF-β signaling, developed spontaneous autoimmune disease (18, 19). IL-10 suppression is mediated via interaction with the IL-10 receptor, expressed on hematopoietic cells, resulting in STAT3 phosphorylation and subsequent activation of a broad range of anti-inflammatory genes. Deficiency in IL-10 leads to spontaneous development of aggressive autoimmune disease in adoptive transfer models of murine colitis. IL-10R polymorphisms have been associated with early-onset UC and impaired TGF-β signaling in IBD patients (20).

### Effector T Cell Subsets

T cells are a major source of pro-inflammatory cytokines in IBD. Gut resident T cells exist in a tolerogenic environment. However, patients with IBD have excessive intestinal T cell activation (21). The cause of this T cell dysregulation is largely unknown. T cells that produce IFNγ have long been implicated in onset of IBD. IFNγ is a pro-inflammatory cytokine vital for immune responses and has been linked to IBD severity in mice and humans (22). IFNγ recruits immune cells to sites of infection and improves inflammatory responses by inducing major histocompatibility complex (MHC) class I and II expression (23). IFNγ directly increases intestinal epithelial permeability by reducing expression of tight junction proteins and perturbing apical actin organization (24). Th17 cells produce IL-17, a pro-inflammatory cytokine with functional roles in many autoimmune diseases, such as rheumatoid arthritis and IBD. T cell populations that express either IFNγ and/or IL-17 are often found at higher frequencies in human inflamed tissues of IBD patients (24, 25). Excessive T cell activation in IBD can be caused by numerous pathways: (1) Inflammatory T cell subsets are resistant to Treg suppression (26); (2) T cells may be specific for commensal bacterial species (27); (3) unregulated activation of APCs in the gut (28); (4) compromised intestinal barrier integrity, leading to recurring bacterial insult (29). It is likely that IBD is just a term for manifestation of gut inflammation, and the mechanisms of disease are much more complex than our current categorization of CD and UC allows. It is also likely, that in many cases of IBD, T cells are mediators of gut inflammation, but not the initial cause of disease. Nonetheless, adoptive transfer murine models of colitis have shown that T cells alone can cause IBD-like pathology, and suppression of T cell pathways can abrogate intestinal inflammation (30).

### Immune Cell Regulation and Function

Polymorphisms in *NOD2* and *ATG16L1* loci have been highly associated with IBD (31). Nucleotide-binding oligomerization domain-containing protein 2 (NOD2) is a pattern recognition receptor expressed by a range of cells, including monocytes, dendritic cells, macrophages, and enterocytes. NOD2 recognizes muramyl dipeptide (MDP), a cell wall protein expressed by both gram-positive and gram-negative bacteria. Recognition of MDP by NOD2 induces a signaling cascade that leads to phosphorylation of IκB, which activates NF-κB, an inducer of inflammatory cytokine responses (32, 33). In healthy people, activation of the NOD2 pathway leads to immune cell activation, generation of pro-inflammatory cytokines, and eventual bacterial clearance (34). Compared to wild-type (WT) mice, *NOD2*-knockout mice have reduced bacterial clearance, reduced numbers of goblet cells, reduced protective mucins and anti-microbial molecules, and increased abundance of non-commensal bacteria, such as *Bacteroides vulgatus—*each of these abnormalities can contribute to intestinal dysbiosis and onset of inflammation (35). Human genome wide association studies (GWAS) have shown between 30 and 50% of Crohn's disease patients have *NOD2* polymorphisms, suggesting NOD2 polymorphisms are a risk factor for disease, but alone, not sufficient or necessary for disease (32, 36). Intestinal organoids offer a valuable model to investigate mechanisms of disease, as the model itself is highly manipulatable, biologically relevant, and suitable for genetic manipulation (3).

The *Atg16l1* gene is highly associated with incidence of Crohn's disease. *Atg16l1* encodes a core structural protein of immune cell autophagosomes (37). Bacteria, bacterial antigens, and cellular components are packaged within intracellular phagosomes for degradation. Phagosomes, loaded with products destined for degradation, fuse with degradative enzyme-containing lysosomes—forming an autophagolysosome (37). In a healthy system, the autophagolysosome facilitates degradation of cellular components into reusable products—macroautophagy. *Atg16l1* polymorphisms have been shown to reduce the formation of phagosomes. Murthy et al. (38) showed that in a CD variant (T316A), murine and primary human macrophages had enhanced degradation of ATG16L1 proteins by Caspase 3, resulting in defective bacterial clearance compared to controls (38). The inability to degrade and recycle cellular and bacterial components can lead to their accumulation. Not only does this starve the immune cell of a vital source of recycled cellular components, accumulation of these products can have potent inflammatory effects upon cell death. High concentrations of cellular debris, both host-derived and foreign, can be recognized as damage-associated molecular patterns (DAMPs) and pathogen-associated molecular patterns (PAMPs), respectively. PAMPs can be recognized by a variety of Pattern Recognition Receptors, and the release of high concentrations of PAMPs from autophagy-deficient cells can be recognized as an infection—generating immune responses by other immune cells. DAMPs are potent “danger-signals” released from damaged tissues and can be recognized by a variety of immune cells, eliciting a potent pro-inflammatory response, leading to pathology and further cell death. Expression of TLR2, TLR4, and TLR9 is higher in inflamed tissue from IBD patients, which could exacerbate inflammatory responses to DAMP and PAMP accumulation (39, 40).

## The Intestinal Organoid Model

The gastrointestinal tract is a complex organ that is constantly exposed to foreign materials and organisms. The intestinal epithelium is a single layer of cells, with entire cell turnover every 2–6 days (41). The stem cells responsible for this continual turnover are positioned within the base of crypts, tube-like invaginations that facilitate the protection of the stem cells from constituents of the luminal environment. The stem cells, which are identified by their expression of the Wnt-target surface protein, Lgr5, persist in a niche defined by the secretions of neighboring cells within the crypts and underlying mesenchymal cells. Intestinal crypt stem cells can be cultured with deep-crypt cell factors in specialized media to mimic the *in vivo* environment. Wnt, produced by both the underlying mesenchymal cells and flanking Paneth cells in the small intestine, is an inducer of Ascl2, a master regulator of stem cell phenotype. Lgr5+ stem cells proliferate and generate transit-amplifying (TA) cells. TA cells are highly proliferative cells that divide a finite number of times before differentiating into gut specialist cells (42). As these cells are generated, they move toward the lumen, distal to the crypt. As Paneth cells are found deep within crypts, the Wnt concentration lessens as TA cells ascend toward the lumen, allowing TA cells to differentiate into their mature cell phenotypes. The large intestine does not contain Wnt-producing Paneth cells, but receives Wnt from the underlying mesenchyme and potentially specialized epithelial cells (43).

Organoid cultures can be established either from individual Lgr5+ stem cells or isolated stem cell-containing crypts. These are seeded into a supporting matrix, such as Matrigel, which provides the high laminin levels characteristic of the stem cell niche (44). Newly established large intestine organoid cultures do not contain functional Wnt-producing cells; therefore, exogenous Wnt is added. In small intestinal organoids, the development of Paneth cells allows a reduction in exogenous Wnt levels, but the absence of mesenchymal and Paneth cells in colonic organoids requires the continued presence of relatively high levels of exogenous Wnt in the media to maintain the cultures. Cell fate is determined by exposure to different niche factors produced by sub-epithelial fibroblasts at the serosal surface of the crypts. Factors such as Noggin and Gremlin inhibit bone morphogenetic protein (BMP), inducing cellular differentiation (45, 46). Differentiation of crypt cells occur in response to the inverse gradient relationship between Wnt and BMP. Wnt concentrations are highest in the base of the crypt, as cells ascend toward the lumen, BMP concentration rises as Wnt concentration falls, inducing differentiation in ascending cells. This crypt design, coupled with mesenchymal and flanking cell chemical influences, allows the IEB to produce cells in a stochastic manner. This complex cell-to-cell signaling enables neutral drift mechanics, ensuring a specific differentiated cell is generated when required and old cells are shed (47).

Intestinal organoids are derived from the isolation and culture of primary stem cells of intestinal crypts or iPSCs. Intact intestinal crypts can be isolated from colonoscopy biopsy samples by Ca^2+^ chelation (48). While there are a number of different protocols for the isolation of the crypts (3, 49) the common feature is the incubation of the biopsy samples in Ca^2+^-free Ringer solution containing ethylenediaminetetraacetic acid and dithiothreitol, followed by mechanical agitation. This results in the isolation of a relatively pure population of intact intestinal crypts containing Lgr5+ stem cells. When cultured in a suitable matrix, such as Matrigel, stem cells within the intestinal crypts survive, avoid anoikis, and proliferate. The crypts then close, form, and develop into a sphere-shaped organoid structure (spheroid). Over the next several days, the cells that comprise the spheroids proliferate and differentiate into gut specialist cells. Lgr5+ stem cells migrate to different areas of the spheroid and undergo crypt-fission events, generating multiple sites of proliferation, thus expanding the culture (3). Mature organoids can then be mechanically disrupted and passaged over months, and maintain genetic similarity to their *in vivo* cell counterparts.

## Organoids as a Tool for Immunological Research

The human intestine is a complex organ with strict organization of structural domains. Current intestinal epithelial models, such as Caco-2 cell-lines (an immortalized cell-line derived from human epithelial colorectal adenocarcinoma cells) (50), are useful for drug absorption screening but do not adequately replicate intestinal structure. Tian et al. (51) demonstrated that mouse-derived organoids could validate pathways of microRNA (MIR31) expression. MIR31 was induced by the presence of either TNF or IL-6, similar to results from a colorectal cancer cell line. These data suggest that organoids can be used in conjunction with cell lines to provide a biologically relevant point of comparison *in vitro*. Organoids allow for in-depth analysis of pathogen-host cell interactions and investigation and validation of human cell mechanisms and pathways. However, in general, current organoid systems do not contain functioning immune cells, nor do they contain microfold (M) cells, a critical cell for intestinal antigen sampling. The introduction of a functional immune system to organoid models could change the way intestinal disease is modeled and investigated.

The human immune system is difficult to study, as samples taken from donors are only a snapshot in time. The ability to culture patient tissues and co-culture with the same patient's immune cells is, in theory, the gold standard for immunological research. In an organoid model, the researcher has control of the cells added to culture, providing the ability to systematically observe the effect each cell type might have on a specific epithelium. For instance, IBD patients often have low frequencies of intestinal CD103+ DCs, and CD103+ DCs promote intestinal tolerance. If patient DCs are isolated, expanded, and induced to a tolerogenic phenotype: can epithelial integrity be restored *in vitro*? How does the addition of specific immune subsets affect organoid permeability and growth? Does a beneficial effect require the addition of a combination of immune subsets? Can the addition of recombinant proteins improve model readouts? How does the addition of a microbiota affect these changes? Can immune cell subsets or cytokines of interest be identified to elucidate the most relevant treatment option for an individual? The organoid model provides a tool to answer these questions by allowing a systematic approach to an overly complex immunological system.

3D organoids contain no stromal tissues or lamina propria. Recently, groups have generated 2D monolayers from 3D organoid culture systems (52). This is an interesting model concept, sacrificing crypt-physiology, but gaining the ability to further manipulate the model system. Mechanically disrupted 3D organoids are seeded on Transwell^®^ membrane plates, generating a polarized gut cell lining that acts as a selectively-permeable barrier, separating the apical and basal compartments of the well. This effectively mimics *in vivo* physiology, providing a lumen (apical compartment) and a lamina propria (basal compartment). This model system has been shown to be effective for the investigation of pathogen-epithelial-immune cell interactions. Noel et al. generated this system using human small intestine organoids co-cultured with macrophages and pathogenic *Escherichia coli* (52). Macrophages were observed extending dendrites through the monolayer to interact with *E. coli* in the “lumen.” Co-culture with macrophages also provided resistance to pathogenic *E. coli*-induced permeability. These data suggest that the organoid model is highly adaptable to experimental requirements, and relevant for the investigation of the immune system in the gut.

M cells endocytose luminal antigens for presentation to immune cells in the lamina propria in a highly controlled manner (53). M cells are positioned in mucosa-associated lymphoid tissues (MALT), areas of dense immune cell presence, found in the submucosal regions of the gastrointestinal tract. M cells sample antigens, enclose them in vesicles, then deliver the vesicles to immune cells stationed directly adjacent to the M cell basolateral membrane. A tolerogenic environment is logical for an environment exposed to high levels of foreign material; however, as a consequence of tolerance, the intestine is susceptible to pathogenic insult. M cells allow for immune surveillance of the lumen without the requirement of a luminal immune presence. M cells are thus vital to immune preparation and stimulation in the gut. In an adoptive transfer model in which mice lacked Spi-B, a transcription factor critical for the development of M cells (*Spib*^−/−^), *Spib*^−^^/-^ mice had reduced bacterial uptake and sampling in Peyer's patches, and, as a consequence, had a reduced immune response compared to WT mice (54). It is possible to generate M cells in murine organoid models using recombinant RANKL protein, an NF-κB ligand that induces the expression of SpiB transcription factor and drives M cell differentiation (55). The induction of functional M cells in human organoid models could improve model biological relevance and provide deeper insights into antigen presentation at the intestinal barrier.

## Organoids as a Tool to Investigate IBD-Induced Intestinal Fibrosis

Sites of inflammation in the intestine are at high risk of developing fibrosis, an excessive buildup of connective tissues. During and after an inflammatory response, damaged intestinal tissues are primarily repaired by intestinal myofibroblasts, among other mesenchymal cells (56). Mesenchymal cells promote the deposition of extracellular matrix (ECM), a network of glycoproteins and collagen, which provide structure and anchoring support to surrounding cells. In IBD, disruption of the fibrogenic process can lead to improper repair of the intestinal barrier, leading to the formation of ulcers or fistulas (57, 58). In contrast, uncontrolled overproduction of ECM can cause a buildup of connective tissues, narrowing the intestine (strictures) (56). The generation of ECM is promoted by immune-mesenchyme crosstalk. Inflammatory cytokines, such as TNF, produced by activated macrophages, can promote or inhibit myofibroblast production of various ECM-degrading metalloproteinases (MMP) (59, 60). In contrast, myofibroblast-derived TGF-β1, also induced by TNF, induces the production of myofibroblast-derived tissue inhibitors of metalloproteinases (TIMP), which inhibit MMP-mediated degradation of ECM (59). Thus, a complex crosstalk of immune and mesenchymal cells is required to maintain healthy restoration on inflammation-induced epithelial damage.

Most current organoid models do not include a mesenchymal component. 3D organoids are a collection of intestinal cells in a Matrigel suspension, usually a single layer thick. Organoid monolayers are typically derived from mechanically disrupted organoids, seeded directly onto a plate or membrane. Rodansky et al. (61), cultured 3D embryonic stem cells which were differentiated into human intestinal organoids. Mature organoids with high mesenchymal cell numbers (compared to other organoids) were selected and used to evaluate the efficacy of the anti-fibrotic drug, spironolactone, *in vitro*. Myofibroblasts in their model were activated by TGF-β1 (as indicated by an increase in the mRNA of pro-fibrotic genes), and subsequently inhibited by the addition of spironolactone. Rodansky et al. demonstrated the clinical and research potential of intestinal organoids as a future model of fibrosis, a model which has been thus far been limited to less biologically relevant models and animal models.

## Development of Tools for Personalized Diagnosis and Treatment Assays

Organoid systems could be designed to support co-culture with any immune cell type. While Noel et al. showed that macrophages were able to function in a 2D organoid system, it is unclear if other immune cells would be successful without antigen presentation via the M cell pathway (52). Regardless, these findings provided evidence that an organoid model can facilitate a functional immune cell component that can both interact with and influence the organoid epithelial barrier.

### Throughput

The design of a high throughput personalized screening system could reduce the impact of non-optimal therapy prescription. Theoretically, a high-throughput system could be designed, in which patient organoids are grown in a 96-well plate and tested with different treatments, immune cell compositions, and microbial species. This model design could lead to the optimization of patient treatments before application, reducing chance of treatment failure. Recently, rectal organoids derived from patients with cystic fibrosis were successfully used to predict patient response to treatment (62). This was particularly important as cystic fibrosis patients with rare genetic mutations are not examined in clinical trials. Modern IBD treatments are designed to target multiple immune mediators, highlighting the value of high-throughput *in vitro* personalized screening for identification of promising treatment targets, in an individual.

Organoid cultures could also be useful in understanding the mechanisms of IBD, before onset of inflammation. In some cases of IBD, Th17 cells are responsible for excessive mucosal inflammation via the release of IL-17A, IL-17F, and the heterodimer IL-17A/F (63). If healthy control or IBD patient organoids are co-cultured with IBD patient Th17 cells, it may be possible to identify whether these cells have deleterious effects on organoid growth, proliferation, or permeability. In the same way, Tregs could be investigated for their ability to suppress other immune cells. This could be further investigated with the addition of a functional microbiota in the apical compartment of the system. The current consensus on IBD implicates a loss of tolerance to gut commensals could be the cause of chronic inflammation; however, the field still lacks the evidence required to define which mechanisms of disease are the cause or a consequence of disease. The organoid model provides a unique opportunity to investigate IBD *in vitro*, potentially providing answers to fundamental unknowns of IBD.

### The Intestinal Microbiota

Multiple groups have introduced bacteria to organoids. For example, microinjection of *Salmonella typhimurium* into the lumen of intestinal organoids induced transcriptional changes relating to cytokine expression patterns (64, 65). After injection into the lumen, *S. typhimurium* penetrated host cell membranes and resided within host-cell vacuoles. These experiments suggest that the organoid model is capable of not only hosting microbial lifecycles, but also responding to their presence. Researchers could introduce a large fraction of the species found within an individual's microbiota via microinjection of microbes isolated from patient-specific fecal material. However, fecal material will only provide live-facultative anaerobes, and the oxygen required for organoid culture will not facilitate obligate anaerobe survival. The addition of antibiotics, pH changes, antivirals, antifungals, recombinant cytokines, probiotics, or immune subsets, could provide valuable insight into responses to the microbiota and improve understanding of intestinal homeostasis.

How the microbiota interacts with immune cells of the intestine remains largely unknown. Gut-resident immune cell subsets maintain gut homeostasis, but little is known about the mechanisms of control. Short chain fatty acid production by gut microbes is important for intestinal barrier homeostasis (66). In a study of IBD patients treated with anti-TNF, patients with remitting IBD, but not non-remitting IBD, had a microbiome more similar to that of healthy people (67). Non-remitting IBD patients had lower expression of intestinal metabolites than remitting IBD patients and healthy controls; butyrate expression was restored in remitting IBD patients.

Probiotics may be an important emerging field for initiating intestinal homeostasis for patients with intestinal diseases; however, due to a lack of knowledge on microbe-barrier-immune cell interactions, probiotics remain ineffective as treatments (68). Theoretically, a high-throughput intestinal organoid system could provide insight into these interactions, allowing the production or selection of biologically relevant probiotics that not only reduce dysbiosis, but promote immune tolerance and homeostasis. To achieve this, researchers could use co-culture experiments in which microbes are introduced to organoid systems containing a functional immune system. Positive readouts could include improvement of epithelial integrity, production of tolerogenic cytokines, immune cell differentiation into tolerogenic phenotypes, or a reduction of pro-inflammatory cytokines.

### Genetic Manipulation of The Organoid Model

Organoid models may provide a powerful model for the use of genetic manipulation tools, which could provide answers to fundamental questions posed by current disease research. Tools such as CRISPR-Cas, transposon mutagenesis, and siRNAs could be used to research the effect of known genetic variants identified by GWAS studies. Mutations in the *NOD2* locus are associated with disease in patients with CD; however, mutations in this locus do not manifest in disease in all individuals with *NOD2* mutations. Using genetic modification tools, mutations could be introduced in a stepwise manner, allowing observation of specific genetic variants and their effects on the system. Genetic tools, such as CRISPR-Cas have already proven to be valuable for the observation of the effects of genetic suppression and over-expression. Organoid models could provide a useful model for the validation of GWAS analyses in human tissues.

Zuo et al. (69), derived intestinal organoids from WT and peroxisome proliferator-activated receptor delta (PPARD)-expressing mice, under control of a villin promotor. This approach generated mice that expressed *ppard* in villin-positive gastric progenitor epithelial cells, and subsequent culture of PPARD-positive murine gastric organoids. PPARD, a nuclear hormone receptor, is upregulated in a variety of cancers, including gastric, breast, and lung cancers. Organoids derived from PPARD-positive mice resulted in tumor growth when injected into immunocompetent mice, whereas WT mouse organoids did not. PPARD-positive mice had higher infiltration of CD45+ immune cells into the gastric tissues, and organoids derived from these tissues secreted more CCL20 and CXCL1, than WT mouse organoids. Thus, Zuo et al. generated an organoid model capable of recapitulating the effects of PPARD, *in vitro*. These models allow for a high degree of experimental manipulation which could be used to investigate numerous other markers of interest for cancer and IBD.

## Clinical Relevance

In recent years, a large variety of treatment options for patients with IBD have become available. However, IBD heterogeneity and treatment variety makes it difficult to identify which treatment option is the optimal choice for an individual. This leads to the administration of treatments on a trial and error basis. Biologics, such as immune response targeting monoclonal antibodies (mAb), have paved the way for more precise manipulation of the immune system.

Anti-TNF biologics, such as Infliximab, Adalimumab, and Golimumab, target and block TNF, a potent inducer of inflammation (70–72). Macrophages are a major source of TNF, however, other cells, such as T cells, also produce TNF (10). Blockade of TNF has been successful in reducing severity of a number of diseases, such as rheumatoid arthritis, ankylosing spondylitis, and psoriasis. However, not all patients with IBD respond to anti-TNF treatment, and 23–46% of responders develop resistance to treatment over time, often due to the development of treatment-targeting antibodies (2, 73). More concerning is the number of primary responders, whom in the absence of treatment-targeting antibodies, still have ineffective responses to treatment over time, suggesting different treatment mechanisms are a requirement in individual patients.

Anti-integrin biologics, such as Natalizumab (α4-integrin) and Vedolizumab (α4β7-integrin) aim to prevent the migration of immune cells to the intestinal mucosa (74, 75). DCs present antigen to T cells in gut MALT, which induces the expression of α4β7-integrin on the T cell surface (76). As α4β7+ T cells circulate in the blood, they can be bound by the cell surface-α4β7 ligand, mucosal vascular addressin cell adhesion molecule 1 (MAdCAM-1). MAdCAM-1-expressing cells are highly localized to intestinal high endothelial venules and facilitate capture and trafficking of circulating α4β7+ to the intestinal mucosa (77). Thus, anti-integrin biologic treatments broadly reduce gut inflammation by reducing intestinal mucosa immune cell presence.

Ustekinumab is an mAb that targets the p40 subunit of IL-12 and IL-23 (78). IL-23 is a pro-inflammatory cytokine that induces the differentiation and survival of Th17 cells. Patients with IBD often have high concentrations of IL-23 in blood and intestinal mucosa (79). Ustekinumab treatment has been effective for patients with high numbers of Th17 cells in the intestinal mucosa and for non-responders to anti-TNF therapy. Recently developed treatment options are Filgotinib and Tofacitinib. Filgotinib and Tofacitinib are Janus Kinase (JAK) inhibitors, which inhibit JAK-Signal transducer and activator of transcription (STAT) signaling pathways.

Each of these treatments comes with variable efficacy and a range of side-effects that reduce patient quality of life. Currently, identification of which treatment is appropriate for an individual is conducted step-wise—increasing time-to-remission, reducing patient quality of life, and increasing cost of treatment and care.

## Conclusions

The gold standard of a scientific model is one that is: amenable to manipulation, robust, biologically relevant, and sustainable. Intestinal diseases and intestinal cell-cell interactions are limited by the accessibility of fresh human tissue. Human colonic and small intestine tissues are difficult to obtain and have a short life span. Organoid systems offer an elegant solution to this problem via the generation of functional organoids from fresh intestinal adult stem cells. The ability to not only maintain, but expand colonic tissue *in vitro* is a revolutionary breakthrough for intestinal research. The organoid model has the potential to become the gold standard of intestinal and immunological research. The ability to observe patient tissue *in vitro* provides a unique opportunity to observe patient tissues before the onset of disease. However, the model requires more data before this type of investigation can be fully realized. Addition of a functional immune system, a complete microbial influence, and the generation of M cells remain to be optimized. Furthermore, the generation of a universal protocol and mainstream organoid media will make the model more accessible for laboratories and clinics looking to adopt the model and provide more accurate comparison of data between laboratories.

## Author Contributions

HA conceived the idea and wrote the manuscript. AB, MS, and RK provided specific input on organoids, gastroenterology, and immunology, respectively. All authors edited the manuscript.

### Conflict of Interest

The authors declare that the research was conducted in the absence of any commercial or financial relationships that could be construed as a potential conflict of interest.

## References

[1] Molodecky NA Soon IS Rabi DM Ghali WA Ferris M Chernoff G. Increasing incidence and prevalence of the inflammatory Bowel diseases with time, based on systematic review. Gastroenterology. (2012) 142:46–54.e42; quiz e30. 10.1053/j.gastro.2011.10.001 22001864

[2] Ding NS Hart A De Cruz P. Systematic review: predicting and optimising response to anti-TNF therapy in Crohn's disease - algorithm for practical management. Aliment Pharmacol Ther. (2016) 43:30–51. 10.1111/apt.13445 26515897

[3] Sato T Stange DE Ferrante M Vries RG Van Es JH Van den Brink S. Long-term expansion of epithelial organoids from human colon, adenoma, adenocarcinoma, and Barrett's epithelium. Gastroenterology. (2011) 141:1762–72. 10.1053/j.gastro.2011.07.050 21889923

[4] Spence JR Mayhew CN Rankin SA Kuhar MF Vallance JE Tolle K. Directed differentiation of human pluripotent stem cells into intestinal tissue *in vitro*. Nature. (2011) 470:105–9. 10.1038/nature09691 21151107 PMC3033971

[5] Sartor RB. Mechanisms of disease: pathogenesis of Crohn's disease and ulcerative colitis. Nat Clin Pract Gastroenterol Hepatol. (2006) 3:390–407. 10.1038/ncpgasthep0528 16819502

[6] Cooney R Baker J Brain O Danis B Pichulik T Allan P. NOD2 stimulation induces autophagy in dendritic cells influencing bacterial handling and antigen presentation. Nat Med. (2010) 16:90–7. 10.1038/nm.2069 19966812

[7] Sobczak M Fabisiak A Murawska N Wesolowska E Wierzbicka P Wlazlowski M. Current overview of extrinsic and intrinsic factors in etiology and progression of inflammatory Bowel diseases. Pharmacol Rep. (2014) 66:766–75. 10.1016/j.pharep.2014.04.005 25149979

[8] Lee J Mo JH Katakura K Alkalay I Rucker AN Liu YT. Maintenance of colonic homeostasis by distinctive apical TLR9 signalling in intestinal epithelial cells. Nat Cell Biol. (2006) 8:1327–36. 10.1038/ncb1500 17128265

[9] Keita AV Salim SY Jiang T Yang PC Franzen L Soderkvist P. Increased uptake of non-pathogenic *E. coli* via the follicle-associated epithelium in longstanding ileal Crohn's disease. J Pathol. (2008) 215:135–44. 10.1002/path.2337 18348161

[10] Sun M He C Cong Y Liu Z. Regulatory immune cells in regulation of intestinal inflammatory response to microbiota. Mucosal Immunol. (2015) 8:969–78. 10.1038/mi.2015.49 26080708 PMC4540654

[11] Cerovic V Houston SA Scott CL Aumeunier A Yrlid U Mowat AM. Intestinal CD103(-) dendritic cells migrate in lymph and prime effector T cells. Mucosal Immunol. (2013) 6:104–13. 10.1038/mi.2012.53 22718260

[12] Iliev ID Spadoni I Mileti E Matteoli G Sonzogni A Sampietro GM. Human intestinal epithelial cells promote the differentiation of tolerogenic dendritic cells. Gut. (2009) 58:1481–9. 10.1136/gut.2008.175166 19570762

[13] Matsuno H Kayama H Nishimura J Sekido Y Osawa H Barman S. CD103+ dendritic cell function is altered in the colons of patients with ulcerative colitis. Inflamm Bowel Dis. (2017) 23:1524–34. 10.1097/MIB.0000000000001204 28700533

[14] Mazmanian SK Liu CH Tzianabos AO Kasper DL. An immunomodulatory molecule of symbiotic bacteria directs maturation of the host immune system. Cell. (2005) 122:107–18. 10.1016/j.cell.2005.05.007 16009137

[15] Breyner NM Michon C de Sousa CS Vilas Boas PB Chain F Azevedo VA. Microbial anti-inflammatory molecule (MAM) from *Faecalibacterium prausnitzii* shows a protective effect on DNBS and DSS-induced colitis model in mice through inhibition of NF-kappaB pathway. Front Microbiol. (2017) 8:114. 10.3389/fmicb.2017.00114 28203226 PMC5285381

[16] Margarita M-M Xavier A Ferran G-H Doroteo A Jesús G-G Abnormal microbiota composition in the ileocolonic mucosa of Crohn's disease patients as revealed by polymerase chain reaction Y denaturing gradient gel electrophoresis. Inflamm Bowel Dis. (2006) 12:1136–45. 10.1097/01.mib.0000235828.09305.0c 17119388

[17] Lamouille S Xu J Derynck R. Molecular mechanisms of epithelial-mesenchymal transition. Nat Rev Mol Cell Biol. (2014) 15:178–96. 10.1038/nrm3758 24556840 PMC4240281

[18] Probert L Keffer J Corbella P Cazlaris H Patsavoudi E Stephens S. Wasting, ischemia, and lymphoid abnormalities in mice expressing T cell-targeted human tumor necrosis factor transgenes. J Immunol. (1993) 151:1894–906. 8345187

[19] Kontoyiannis D Pasparakis M Pizarro TT Cominelli F Kollias G. Impaired on/off regulation of TNF biosynthesis in mice lacking TNF AU-rich elements: implications for joint and gut-associated immunopathologies. Immunity. (1999) 10:387–98. 10.1016/S1074-7613(00)80038-2 10204494

[20] Moran CJ Walters TD Guo C-H Kugathasan S Klein C Turner D. IL-10R polymorphisms are associated with very-early-onset ulcerative colitis. Inflamm Bowel Dis. (2012) 19:115–23. 10.1002/ibd.22974 22550014 PMC3744177

[21] Duchmann R Kaiser I Hermann R Mayet W Ewe K Meyer Zum Büschenfelde K. Tolerance exists towards resident intestinal flora but is broken in active inflammatory Bowel disease.pdf. Clin Exp Immunol. (1995) 102:448–55. 10.1111/j.1365-2249.1995.tb03836.x 8536356 PMC1553362

[22] Fuss IJ Neurath M Boirivant M Klein JS de la Motte C Strong SA. Disparate CD4+ lamina propria (LP) lymphokine secretion profiles in inflammatory Bowel disease. Crohn's disease LP cells manifest increased secretion of IFN-gamma, whereas ulcerative colitis LP cells manifest increased secretion of IL-5. J Immunol. (1996) 157:1261–70. 10.1097/00024382-199703001-00528 8757634

[23] Rosa F Fellous M. Regulation of HLA-DR gene by IFN-gamma. Transcriptional and post-transcriptional control. J Immunol. (1988) 140:1660–4. 3126233

[24] Youakim A Ahdieh M. Interferon-γ decreases barrier function in T84 cells by reducing ZO-1 levels and disrupting apical actin. Am J Physiol. (1999) 276:G1279–88. 10.1152/ajpgi.1999.276.5.G1279 10330020

[25] Jiang W Su J Zhang X Cheng X Zhou J Shi R. Elevated levels of Th17 cells and Th17-related cytokines are associated with disease activity in patients with inflammatory Bowel disease. Inflamm Res. (2014) 63:943–50. 10.1007/s00011-014-0768-7 25129403

[26] Goodman WA Young AB McCormick TS Cooper KD Levine AD. Stat3 phosphorylation mediates resistance of primary human T cells to regulatory T cell suppression. J Immunol. (2011) 186:3336–45. 10.4049/jimmunol.1001455 21307288 PMC3133678

[27] Hepworth MR Fung TC Masur SH Kelsen JR McConnell FM Dubrot J. Group 3 innate lymphoid cells mediate intestinal selection of commensal bacteria-specific CD4(+) T cells. Science. (2015) 348:1031–5. 10.1126/science.aaa4812 25908663 PMC4449822

[28] Rogler G Hausmann M Spottl T Vogl D Aschenbrenner E Andus T. T-cell co-stimulatory molecules are upregulated on intestinal macrophages from inflammatory Bowel disease mucosa. Eur J Gastroenterol Hepatol. (1999) 11:1105–11. 10.1097/00042737-199910000-00006 10524639

[29] Clayburgh DR Shen L Turner JR. A porous defense: the leaky epithelial barrier in intestinal disease. Lab Invest. (2004) 84:282–91. 10.1038/labinvest.3700050 14767487

[30] Elson CO Cong Y Weaver CT Schoeb TR McClanahan TK Fick RB. Monoclonal anti-interleukin 23 reverses active colitis in a T cell-mediated model in mice. Gastroenterology. (2007) 132:2359–70. 10.1053/j.gastro.2007.03.104 17570211

[31] Gazouli M Pachoula I Panayotou I Mantzaris G Chrousos G Anagnou NP. NOD2/CARD15, ATG16L1 and IL23R gene polymorphisms and childhood-onset of Crohn's disease. World J Gastroenterol. (2010) 16:1753–8. 10.3748/wjg.v16.i14.1753 20380008 PMC2852824

[32] Hugot J-P Chamaillard M Zouali H Lesage S Cézard J-P Belaiche J. Association of NOD2 leucine-rich repeat variants with susceptibility to Crohn's disease. Nature. (2001) 411:599. 10.1038/35079107 11385576

[33] Ogura Y Bonen D Inohara N Nicolae D Chen F Ramos R. A frameshift mutation in NOD2 associated with susceptibility to Crohn's disease. Lett Nat. (2001) 411:603–6. 10.1038/35079114 11385577

[34] Homer CR Richmond AL Rebert NA Achkar JP McDonald C. ATG16L1 and NOD2 interact in an autophagy-dependent antibacterial pathway implicated in Crohn's disease pathogenesis. Gastroenterology. (2010) 139:1630–41.e1-2. 10.1053/j.gastro.2010.07.006 20637199 PMC2967588

[35] Ramanan D Tang MS Bowcutt R Loke P Cadwell K. Bacterial sensor Nod2 prevents inflammation of the small intestine by restricting the expansion of the commensal *Bacteroides vulgatus*. Immunity. (2014) 41:311–24. 10.1016/j.immuni.2014.06.015 25088769 PMC4238935

[36] Yamamoto S Ma X. Role of Nod2 in the development of Crohn's disease. Microbes Infect. (2009) 11:912–8. 10.1016/j.micinf.2009.06.005 19573617 PMC2924159

[37] Xie Z Klionsky DJ. Autophagosome formation: core machinery and adaptations. Nat Cell Biol. (2007) 9:1102. 10.1038/ncb1007-1102 17909521

[38] Murthy A Li Y Peng I Reichelt M Katakam AK Noubade R. A Crohn's disease variant in Atg16l1 enhances its degradation by caspase 3. Nature. (2014) 506:456–62. 10.1038/nature13044 24553140

[39] Hausmann M Kiessling S Mestermann S Webb G Spöttl T Andus T. Toll-like receptors 2 and 4 are up-regulated during intestinal inflammation. Gastroenterology. (2002) 122:1987–2000. 10.1053/gast.2002.33662 12055604

[40] Sánchez-Muñoz F Fonseca-Camarillo GC Villeda-Ramirez MA Barreto-Zuniga R Bojalil R Domínguez-Lopez A. TLR9 mRNA expression is upregulated in patients with active ulcerative colitis. Inflamm Bowel Dis. (2009) 16:1267–8. 10.1002/ibd.21155 19902548

[41] Williams JM Duckworth CA Burkitt MD Watson AJ Campbell BJ Pritchard DM. Epithelial cell shedding and barrier function: a matter of life and death at the small intestinal villus tip. Vet Pathol. (2015) 52:445–55. 10.1177/0300985814559404 25428410 PMC4441880

[42] Schuijers J Junker JP Mokry M Hatzis P Koo BK Sasselli V. Ascl2 acts as an R-spondin/Wnt-responsive switch to control stemness in intestinal crypts. Cell Stem Cell. (2015) 16:158–70. 10.1016/j.stem.2014.12.006 25620640

[43] Degirmenci B Valenta T Dimitrieva S Hausmann G Basler K. GLI1-expressing mesenchymal cells form the essential Wnt-secreting niche for colon stem cells. Nature. (2018) 558:449–53. 10.1038/s41586-018-0190-3 29875413

[44] Kleinman HK Martin GR. Matrigel: basement membrane matrix with biological activity. Semin Cancer Biol. (2005) 15:378–86. 10.1016/j.semcancer.2005.05.004 15975825

[45] Hsu DR Economides AN Wang X Eimon PM Harland RM. The Xenopus dorsalizing factor Gremlin identifies a novel family of secreted proteins that antagonize BMP activities. Mol Cell. (1998) 1:673–83. 10.1016/S1097-2765(00)80067-2 9660951

[46] Qi Z Li Y Zhao B Xu C Liu Y Li H. BMP restricts stemness of intestinal Lgr5(+) stem cells by directly suppressing their signature genes. Nat Commun. (2017) 8:13824. 10.1038/ncomms13824 28059064 PMC5227110

[47] Crosnier C Stamataki D Lewis J. Organizing cell renewal in the intestine: stem cells, signals and combinatorial control. Nat Rev Genet. (2006) 7:349–59. 10.1038/nrg1840 16619050

[48] Mahe MM Aihara E Schumacher MA Zavros Y Montrose MH Helmrath MA. Establishment of gastrointestinal epithelial organoids. Curr Protoc Mouse Biol. (2013) 3:217–40. 10.1002/9780470942390.mo130179 25105065 PMC4120977

[49] O'Rourke KP Ackerman S Dow LE Lowe SW. Isolation, culture, and maintenance of mouse intestinal stem cells. Bio Protoc. (2016) 6:e1733. 10.21769/BioProtoc.1733 27570799 PMC4996657

[50] Hidalgo IJ Raub TJ Borchardt RT. Characterization of the human colon carcinoma cell line (Caco-2) as a model system for intestinal epithelial permeability. Gastroenterology. (1989) 96:736–49. 10.1016/S0016-5085(89)80072-1 2914637

[51] Tian Y Xu J Li Y Zhao R Du S Lv C. MicroRNA-31 reduces inflammatory signaling and promotes regeneration in colon epithelium, and delivery of mimics in microspheres reduces colitis in mice. Gastroenterology. (2019) 156:2281–96.e2286. 10.1053/j.gastro.2019.02.023 30779922

[52] Noel G Baetz NW Staab JF Donowitz M Kovbasnjuk O Pasetti MF A primary human macrophage-enteroid co-culture model to investigate mucosal gut physiology and host-pathogen interactions. Sci Rep. (2017) 7:45270 10.1038/srep45270 28345602 PMC5366908

[53] Mabbott NA Donaldson DS Ohno H Williams IR Mahajan A. Microfold (M) cells: important immunosurveillance posts in the intestinal epithelium. Mucosal Immunol. (2013) 6:666–77. 10.1038/mi.2013.30 23695511 PMC3686595

[54] Kanaya T Hase K Takahashi D Fukuda S Hoshino K Sasaki I. The Ets transcription factor Spi-B is essential for the differentiation of intestinal microfold cells. Nat Immunol. (2012) 13:729–36. 10.1038/ni.2352 22706340 PMC3704196

[55] Rouch JD Scott A Lei NY Solorzano-Vargas RS Wang J Hanson EM. Development of functional microfold (M) cells from intestinal stem cells in primary human enteroids. PLoS ONE. (2016) 11:e0148216. 10.1371/journal.pone.0148216 26820624 PMC4731053

[56] Lenti MV Di Sabatino A. Intestinal fibrosis. Mol Aspects Med. (2019) 65:100–9. 10.1016/j.mam.2018.10.003 30385174

[57] Bataille F Klebl F Rümmele P Schroeder J Farkas S Wild P. Morphological characterisation of Crohn's disease fistulae. Gut. (2004) 53:1314–21. 10.1136/gut.2003.038208 15306592 PMC1774207

[58] Scharl M What distinguishes mechanisms of fistula and stricture formation. In: Rieder F editor. Fibrostenotic Inflammatory Bowel Disease. Cham: Springer (2018). p. 307–17.

[59] Theiss AL Simmons JG Jobin C Lund PK. Tumor necrosis factor (TNF) α increases collagen accumulation and proliferation in intestinal myofibroblasts via TNF receptor 2. J Biol Chem. (2005) 280:36099–109. 10.1074/jbc.M505291200 16141211

[60] Tsai C-L Chen W-C Hsieh H-L Chi P-L Hsiao L-D Yang C-M. TNF-α induces matrix metalloproteinase-9-dependent soluble intercellular adhesion molecule-1 release via TRAF2-mediated MAPKs and NF-κB activation in osteoblast-like MC3T3-E1 cells. J Biomed Sci. (2014) 21:12. 10.1186/1423-0127-21-12 24502696 PMC3926355

[61] Rodansky ES Johnson LA Huang S Spence JR Higgins PD. Intestinal organoids: a model of intestinal fibrosis for evaluating anti-fibrotic drugs. Exp Mol Pathol. (2015) 98:346–51. 10.1016/j.yexmp.2015.03.033 25828392 PMC5915372

[62] Berkers G van Mourik P Vonk AM Kruisselbrink E Dekkers JF de Winter-de Groot KM. Rectal organoids enable personalized treatment of cystic fibrosis. Cell Rep. (2019) 26:1701–8.e1703. 10.1016/j.celrep.2019.01.068 30759382

[63] Galvez J. Role of Th17 cells in the pathogenesis of human IBD. ISRN Inflamm. (2014) 2014:928461. 10.1155/2014/928461 25101191 PMC4005031

[64] Forbester JL Goulding D Vallier L Hannan N Hale C Pickard D. Interaction of *Salmonella enterica* Serovar Typhimurium with intestinal organoids derived from human induced pluripotent stem cells. Infect Immun. (2015) 83:2926–34. 10.1128/IAI.00161-15 25964470 PMC4468523

[65] Wilson SS Tocchi A Holly MK Parks WC Smith JG. A small intestinal organoid model of non-invasive enteric pathogen-epithelial cell interactions. Mucosal Immunol. (2015) 8:352–61. 10.1038/mi.2014.72 25118165 PMC4326599

[66] Lazar V Ditu LM Pircalabioru G Gheorghe I Curutiu C. Aspects of gut microbiota and immune system interactions in infectious diseases, immunopathology and cancer. Front Immunol. (2018) 9:1830. 10.3389/fimmu.2018.01830 30158926 PMC6104162

[67] Aden K Rehman A Waschina S Pan W-H Walker A Lucio M. Metabolic functions of gut microbes associate with efficacy of tumor necrosis factor antagonists in patients with inflammatory Bowel diseases. Gastroenterology. (2019) 157:1279–92.e1211. 10.1053/j.gastro.2019.07.025 31326413

[68] Sanders ME Merenstein DJ Reid G Gibson GR Rastall RA Probiotics and prebiotics in intestinal health and disease: from biology to the clinic. Nat Rev Gastroenterol Hepatol. (2019) 6:605–16. 10.1038/s41575-019-0173-3. 31296969

[69] Zuo X Deguchi Y Xu W Liu Y Li HS Wei D. PPARD and Interferon gamma promote transformation of gastric progenitor cells and tumorigenesis in mice. Gastroenterology. (2019) 157:163–78. 10.1053/j.gastro.2019.03.018 30885780 PMC6581611

[70] Hanauer SB Feagan BG Lichtenstein GR Mayer LF Schreiber S Colombel JF. Maintenance infliximab for Crohn's disease: the ACCENT I randomised trial. Lancet. (2002) 359:1541–9. 10.1016/S0140-6736(02)08512-4 12047962

[71] Bartelds GM Wijbrandts CA Nurmohamed MT Stapel S Lems WF Aarden L. Clinical response to adalimumab: relationship to anti-adalimumab antibodies and serum adalimumab concentrations in rheumatoid arthritis. Ann Rheum Dis. (2007) 66:921–6. 10.1136/ard.2006.065615 17301106 PMC1955110

[72] Sandborn WJ Feagan BG Marano C Zhang H Strauss R Johanns J. Subcutaneous golimumab induces clinical response and remission in patients with moderate-to-severe ulcerative colitis. Gastroenterology. (2014) 146:85–95; quiz e14–85. 10.1053/j.gastro.2013.05.048 23735746

[73] Baert F Noman M Vermeire S Van Assche G D'haens G Carbonez A. Influence of immunogenicity on the long-term efficacy of infliximab in Crohn's disease. N Engl J Med. (2003) 348:601–8. 10.1056/NEJMoa020888 12584368

[74] Selewski DT Shah GV Segal BM Rajdev PA Mukherji SK. Natalizumab (Tysabri). AJNR Am J Neuroradiol. (2010) 31:1588–90. 10.3174/ajnr.A2226 20688889 PMC7964985

[75] Wyant T Fedyk E Abhyankar B. An overview of the mechanism of action of the monoclonal antibody Vedolizumab. J Crohns Colitis. (2016) 10:1437–44. 10.1093/ecco-jcc/jjw092 27252400

[76] Stagg AJ Kamm MA Knight SC. Intestinal dendritic cells increase T cell expression of α4β7 integrin. Eur J Immunol. (2002) 32:1445–54. 10.1002/1521-4141(200205)32:5<1445::AID-IMMU1445>3.0.CO;2-E 11981833

[77] Berlin C Berg EL Briskin MJ Andrew DP Kilshaw PJ Holzmann B α4β7 integrin mediates lymphocyte binding to the mucosal vascular addressin MAdCAM-1. Cell. (1993) 74:185–95. 10.1016/0092-8674(93)90305-A 7687523

[78] Benson JM Peritt D Scallon BJ Heavner GA Shealy DJ Giles-Komar JM. Discovery and mechanism of ustekinumab: a human monoclonal antibody targeting interleukin-12 and interleukin-23 for treatment of immune-mediated disorders. MAbs. (2011) 3:535–45. 10.4161/mabs.3.6.17815 22123062 PMC3242840

[79] Liu Z Yadav PK Xu X Su J Chen C Tang M. The increased expression of IL-23 in inflammatory Bowel disease promotes intraepithelial and lamina propria lymphocyte inflammatory responses and cytotoxicity. J Leukoc Biol. (2011) 89:597–606. 10.1189/jlb.0810456 21227898

